# Systematic analysis of proton pump inhibitors-related adverse reactions using the FDA adverse event reporting system database

**DOI:** 10.1371/journal.pone.0340684

**Published:** 2026-02-02

**Authors:** Zhenyu Wang, Jianan Jin, Guimei Wang, Guoqi Zhou, Hanliang Jiang

**Affiliations:** 1 Pulmonary and Critical Care Medicine, Regional medical center for National Institute of Respiratory Disease, Sir Run Run Shaw Hospital, School of Medicine, Zhejiang University, Hangzhou, Zhejiang, P. R. China; 2 School of Medicine, Shaoxing university, Shaoxing, Zhejiang, People's Republic of China; 3 Graduate School, Zhejiang Chinese Medical University, People's Republic of China; 4 Pulmonary Disease Department, Zunyi of Traditional Hospital Chinese Medicine, Zunyi, Guizhou, People's Republic of China; University of Naples Federico II: Universita degli Studi di Napoli Federico II, ITALY

## Abstract

**Background:**

Proton pump inhibitors (PPIs), known for their potent acid-suppressing effects, are widely used in various clinical settings, including treatment and prevention. Understanding their adverse effects is crucial. This study, utilizing the FDA Adverse Event Reporting System (FAERS) database, comprehensively analyzes PPI-related adverse events to guide clinical medication practices.

**Methods:**

This study analyzed suspected adverse drug reactions (ADRs) related to specific PPI drugs using data from the FAERS database, covering Q1 2004 to Q4 2024. Multiple statistical methods, including ROR, PRR, IC025, and EBGM, were employed for evaluation, with ADRs defined according to System Organ Class (SOC) and Preferred Term (PT). A comparative analysis was conducted to assess potential differences in ADR profiles among different PPI drugs.

**Results:**

This study analyzed 176,680 cases of PPI-related adverse events, with a total of 632,468 adverse reaction reports recorded when PPIs were designated as the primary suspected drug (PS). PPIs showed significantly elevated risks in the renal/urinary and gastrointestinal systems, with other common adverse reactions including hypomagnesemia, hypocalcemia, and renal anemia. Most adverse reactions occurred either within the first 0–30 days of use or after prolonged exposure (>6 months), and elderly patients (≥65 years) were disproportionately affected.

**Conclusions:**

For high-risk populations using PPIs long-term (such as elderly patients or those with pre-existing renal impairment), continuous monitoring is essential to mitigate potential complications. Unnecessary use should be strictly avoided, and long-term medication should be minimized to ensure safety and appropriateness.

## 1. Introduction

PPIs are among the most widely prescribed medications globally. Their potent acid-suppressive effects make them a mainstay in the management of acid-related disorders such as gastroesophageal reflux disease (GERD), peptic ulcer disease (PUD), and Helicobacter pylori eradication therapy [1,2]. Furthermore, PPIs are extensively utilized for preventing gastrointestinal complications associated with long-term use of certain medications, including nonsteroidal anti-inflammatory drugs (NSAIDs), anticoagulants, and corticosteroids [3,4]. Studies have consistently documented a high prevalence of off-label PPI use worldwide, encompassing both inappropriate utilization without valid indications and prolonged maintenance therapy without dose reduction despite established indications. Although post-marketing surveillance generally supports the safety and efficacy of PPIs as acid-suppressive agents, an increasing number of adverse events related to their long-term administration have been reported [5–7]. Growing research attention is now directed toward potential safety concerns associated with chronic PPI use. Evidence from case reports and cohort studies continues to deepen our understanding of both the clinical manifestations and underlying mechanisms of PPI-associated adverse reactions.

PPIs exert their therapeutic effects by irreversibly inhibiting the H ⁺ /K ⁺ -ATPase enzyme in parietal cells, thereby substantially reducing gastric acid secretion. This mechanism not only alleviates symptoms of acid-related disorders but also promotes gastric mucosal healing, solidifying their pivotal role in gastroenterological therapeutics. However, the pharmacodynamic action of acid suppression carries inherent risks [8]. Prolonged gastric acid inhibition may disrupt gastrointestinal microbiome homeostasis, impair nutrient absorption, and interfere with other physiological processes, potentially leading to adverse outcomes such as renal injury, hypomagnesemia, increased fracture risk, heightened susceptibility to infections, and cardiovascular events [9,10]. These multifaceted adverse effects have been extensively investigated and reported, further amplifying concerns about PPI overuse and underscoring the imperative for judicious clinical application [11].

Pharmacovigilance databases possess unique advantages in post-marketing drug safety surveillance. These systems enable timely detection of medication-related safety issues through continuous monitoring, thereby mitigating potential risks. While previous studies utilizing FDA databases have analyzed ADRs associated with specific PPIs – such as Chen et al.‘s investigation highlighting concerns regarding PPI use in patients predisposed to acute kidney injury (AKI) [12], Zhai’s identification of PPIs potentially inducing various cardiac and vascular events (CVEs) [13], Sun’s detection of significant rhabdomyolysis signals [14], and Zhang’s exploration of potential oncogenic risks – the absence of standardized ADR assessment criteria has limited their ability to provide comprehensive comparative analyses [15]. The growing demand for precision medicine and personalized therapy underscores the critical need for holistic understanding of these pharmacological variations, a gap our research aims to address. Furthermore, the continuous accumulation of adverse event data necessitates updated investigations to elucidate emerging ADR patterns and validate previous findings. Consequently, systematic characterization of PPI-associated adverse reactions and their underlying mechanisms is imperative for optimizing clinical pharmacotherapy.

This study conducted a systematic retrospective analysis of PPI-associated adverse reactions using data mining techniques applied to the FAERS database. Through quantitative disproportionality analyses including reporting odds ratio (ROR), proportional reporting ratio (PRR), information component (IC025), and empirical Bayesian geometric mean (EBGM), we investigated the potential risks and associations of PPI-related ADRs. Furthermore, comparative analyses of inter-PPI pharmacovigilance profiles were performed to elucidate class-specific versus agent-specific safety concerns, thereby providing evidence-based insights for optimizing therapeutic decision-making and enhancing medication safety in clinical practice.

## 2. Materials and methods

### 2.1. Data source

This study employed the FAERS database, which contains over 20 million ADR reports from the United States, Europe, and Asia. Reports are submitted by both healthcare professionals (including physicians, pharmacists, and registered nurses) and non-healthcare individuals (such as consumers, lawyers, and vendors). Each report provides a unique case identifier, along with patient demographic details, report date, reporting country, qualifications of the primary reporter, suspected drugs and their indications, ADR occurrence date, severity level, and detailed ADR descriptions. To standardize ADR reporting, each event is coded using PT from the Medical Dictionary for Regulatory Activities (MedDRA) 27.0, with classifications organized according to SOC.

PPIs were approved for clinical use in the late 1980s, and have since experienced significant global market expansion. This study adopted a retrospective pharmacovigilance study design and analyzed FAERS-reported ADRs related to PPIs from Q1 2004 to Q4 2024, covering six active ingredients: esomeprazole, lansoprazole, omeprazole, pantoprazole, rabeprazole, and dexlansoprazole. Both brand and generic names were included, and data underwent deduplication and filtering following FDA recommendations. Data cleaning followed a standardized deduplication rule recommended by the FDA: reports were sorted using CASEID, FDA_DT, and PRIMARYID from the DEMO table. Among reports with identical CASEID, the one with the latest FDA_DT was retained. If both CASEID and FDA_DT were identical, the report with the highest PRIMARYID was preserved. Further cleaning was applied by excluding records with missing or implausible values for key variables such as age (0–120 years), gender (only “Male” or “Female”), and weight (20–200 kg), resulting in a finalized cleaned dataset. To minimize potential confounding from concomitant medications, combination therapies, such as PPIs co-administered with antibiotics, were excluded to reduce false-positive signals. Additionally, reports involving non-drug-related factors, including procedural complications, product issues, and socio-environmental influences, were removed to ensure that only genuine ADR signals were retained. Two independent researchers conducted data processing and validation. In cases of discrepancy, a third researcher reviewed the data and provided the final determination. A targeted literature review was also conducted to contextualize the findings and compare with existing evidence on renal adverse effects of PPIs.

### 2.2. Data analysis

Following data cleaning and deduplication, a total of 176,680 cases involving PPI-related adverse events were included in this study. The analysis covered six major PPIs: Esomeprazole, Lansoprazole, Dexlansoprazole, Omeprazole, Pantoprazole, and Rabeprazole. When PPIs were designated as the primary suspect drug (PS), a total of 632,468 adverse reaction reports were identified. To assess the disproportionate reporting of adverse events, we conducted disproportionality analysis, a widely used method in pharmacovigilance. The following statistical approaches were applied [16,17]:

ROR measures the strength of association between a drug and an adverse event by comparing the frequency of the event in the target drug group to its frequency in the remaining dataset. A ROR lower limit (ROR025) exceeding 1 is considered a positive signal. PRR compares the proportion of reports for a specific adverse event associated with a drug against the proportion for all other drugs. A PRR ≥ 2, χ² ≥ 4, and a minimum of 3 cases indicate a potential signal. Information Component (IC025) – Based on Bayesian statistics, IC quantifies the deviation of observed event frequencies from the expected values. A lower limit of the 95% confidence interval (IC025) > 0 is considered a significant signal. EBGM is a Bayesian method that smooths probability estimates to control for small sample sizes. A lower bound (EB05) exceeding 1 is indicative of a signal.

To ensure the robustness of signal detection, an adverse event was considered a true positive signal only if all four methods yielded positive results. All statistical analyses were conducted using Excel (Microsoft Office 365) and R (version 4.4.2) for data processing, visualization, and statistical calculations.

## 3. Results

### 3.1. Study population

From Q1 2004 to Q4 2024, after data cleaning and deduplication, a total of 176,680 cases of PPIs were reported as the primary PS were included in this study, encompassing 632,468 adverse events. Among these, serious adverse events accounted for 71.8% (n = 126,945), while fatal cases constituted 6.2% (n = 11,005). After excluding cases with missing demographic information, female patients (n = 88,745, 50.2%) accounted for a significantly higher proportion of ADR reports than males (n = 54,313, 30.7%). The age distribution was predominantly middle-aged (45–64 years, 24.4%) and elderly (≥65 years, 25.3%). Geographically, the United States contributed the highest proportion of cases (68.18%), with consumers being the primary reporters (38.6%). Detailed information is presented in Table 1.

**Table 1:** Basic characteristics of PPIs in FAERS.

Characteristic	Esomeprazole (%)	Lansoprazole (%)	Dexlansoprazole (%)	Omeprazole (%)	Pantoprazole (%)	Rabeprazole (%)	Total
Total cases	69438	27461	5882	38783	31216	3900	176680
**Gender**							
Female	39388(56.72)	9709(35.36)	2188(37.20)	20036(51.66)	15645(50.12)	1779(45.62)	88745(50.23)
Male	20425(29.41)	6686(24.35)	973(16.54)	13868(35.76)	11187(35.84)	1174(30.10)	54313(30.74)
Missing	9625(13.86)	11066(40.30)	2721(46.26)	4879(12.58)	4384(14.04)	947(24.28)	33622(19.03)
**Age(years)**							
< 18	687(0.99)	518(1.89)	13(0.22)	1180(3.04)	337(1.08)	26(0.67)	2761(1.56)
18-44	5932(8.54)	1382(5.03)	237(4.03)	4226(10.90)	3135(10.04)	433(11.10)	15345(8.69)
45–64	21527(31.00)	3415(12.44)	655(11.14)	8936(23.04)	7858(25.17)	787(20.18)	43178(24.44)
≥65	16256(23.41)	5486(19.98)	636(10.81)	12066(31.11)	9177(29.40)	1128(28.92)	44749(25.33)
Not Specified	25036(36.06)	16660(60.67)	4341(73.80)	12375(31.91)	10709(34.31)	1526(39.13)	70647(39.99)
**Weight(kg)**							
Mean(SD)	81.75(25.27)	73.54(25.83)	76.45(20.12)	76.61(27.77)	75.19(23.42)	71.20(21.76)	78.28(25.74)
Median(Q1,Q3)	79.40 (65.80,95.00)	71.00 (58.96,86.17)	73.92 (62.13,87.75)	75.00 (62.80,90.00)	73.00 (61.22,86.26)	69.00 (57.15,82.55)	76.25(63.40, 91.08)
N(Missing)	22474(46964)	4684(22777)	1311(4571)	12895(25888)	7747(23469)	1115(2785)	50226(126454)
**Reporters**							
Consumer	31069(44.74)	11410(41.55)	2386(40.56)	14515(37.43)	8307(26.61)	667(17.10)	68354(38.69)
Unspecified	25061(36.09)	1116(4.06)	205(3.49)	6644(17.13)	765(2.45)	180(4.62)	33971(19.23)
Lawyer	2387(3.44)	5060(18.43)	1747(29.70)	784(2.02)	6715(21.51)	810(20.77)	17503(9.91)
Physician	6536(9.41)	3310(12.05)	665(11.31)	6057(15.62)	6099(19.54)	991(25.41)	23658(13.39)
Pharmacist	2476(3.57)	3216(11.71)	332(5.64)	5642(14.55)	5089(16.30)	787(20.18)	17542(9.93)
Other health professional	1909(2.75)	3349(12.20)	547(9.30)	5141(13.26)	4241(13.59)	465(11.92)	15652(8.86)
**Report countries**							
USA	58669(84.49)	18908(68.85)	5434(92.38)	19860(51.21)	15967(51.15)	1632(41.85)	120470 (68.18)
France	3983(5.74)	2086(7.60)	0(0.00)	2152(5.55)	4132(13.24)	525(13.46)	12878 (7.29)
Japan	924(1.33)	1342(4.89)	0(0.00)	457(1.18)	2(0.01)	440(11.28)	3165 (1.79)
Canada	757(1.09)	227(0.83)	109(1.85)	550(1.42)	2363(7.57)	226(5.79)	4232 (2.40)
United Kingdom	701(1.01)	2947(10.73)	0(0.00)	7731(19.93)	444(1.42)	62(1.59)	11885 (6.73)
Other countries	4404(6.34)	1950(7.10)	339(5.76)	8033(20.71)	8308(26.61)	1015(26.03)	24049 (13.61)
**Serious outcomes**							
Death	3690(5.31)	2468(8.99)	499(8.48)	1841(4.75)	2137(6.85)	370(9.49)	11005(6.23)
Hospitalization-initial/prolonged	12376(17.82)	5020(18.28)	439(7.46)	10396(26.81)	8833(28.30)	1135(29.10)	38199(21.62)
Life-threatening	1118(1.61)	671(2.44)	90(1.53)	1936(4.99)	1286(4.12)	107(2.74)	5208(2.95)
Disability	1829(2.63)	478(1.74)	77(1.31)	1573(4.06)	538(1.72)	89(2.28)	4584(2.59)
Other serious outcomes	33021(47.55)	20574(74.92)	3964(67.39)	18955(48.87)	21185(67.86)	1717(44.02)	99416(56.27)

**Fig 1:**
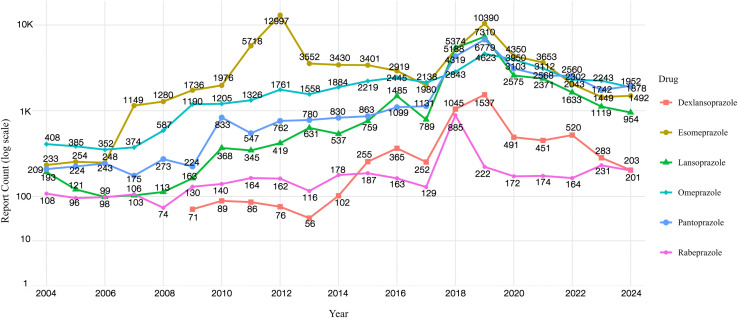
PPI adverse reaction reports(2004-2024). The x-axis shows the reporting year, and the y-axis displays the number of reports on a log10 scale (1, 10, 100, 1k, 10k), illustrating the yearly volume of adverse event submissions related to proton pump inhibitors.

As illustrated in (Fig 1), the number of adverse event reports associated with six PPIs has shown a consistent upward trend over the past two decades (2004–2024). Among these, Dexlansoprazole was approved by the FDA in 2009, and its adverse event reports have been recorded since then. The other five PPIs reached their peak reporting volume in 2019, whereas Rabeprazole peaked earlier in 2018. Esomeprazole exhibited a secondary peak in 2012. This increasing trend may be attributed to multiple factors, including the continuous improvement of the FAERS database, the strengthening of pharmacovigilance systems, the growing prescription volume of PPIs, and the widespread availability of OTC formulations. Additionally, heightened public awareness regarding potential PPI-related adverse events and the intensification of drug safety monitoring have likely further propelled the rise in reported cases.

### 3.2. Primary analysis

**Fig 2:**
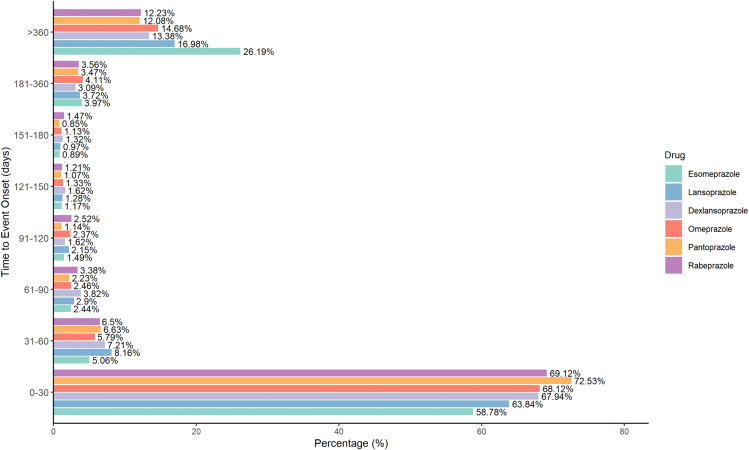
Relationship between the number of adverse reaction reports related to PPIs and the duration of medication. The x-axis lists individual PPIs, and the stacked bars represent the proportion of reports falling into different time-to-onset intervals (0–30 days, 31–90 days, 91–180 days, 181–360 days, > 360 days), allowing visual comparison of onset-time patterns across drugs.

The adverse event reporting pattern associated with PPIs exhibits a distinct time-dependent trend. As shown in (Fig 2), the highest incidence of adverse event reports occurs within the first 0–30 days of PPI use, accounting for more than half of the total reports. This suggests that the risk of adverse reactions is particularly elevated during the initial phase of treatment, necessitating heightened clinical vigilance. In the initial stages of therapy, adjustments should be made promptly based on patient feedback to minimize the occurrence of adverse events. Over time, the reporting rate gradually declines. However, a resurgence in adverse event reports is observed after six months (>180 days), culminating in a second peak during long-term use (>360 days). This pattern highlights the potential for cumulative effects and delayed-onset adverse reactions with prolonged PPI therapy, emphasizing the need for continuous monitoring throughout treatment to mitigate long-term risks.

Esomeprazole demonstrates a distinct pattern compared to other PPIs. During short-term use (0–30 days), its adverse event reporting rate is lower than that of other PPIs, which may be attributed to its pharmacokinetic advantages as an isomer of Omeprazole. Studies indicate that Esomeprazole exhibits a higher area under the curve (AUC) with lower interindividual variability compared to Omeprazole, potentially contributing to a more stable acid-suppressive effect in the short term and consequently reducing the incidence of adverse reactions. Furthermore, Esomeprazole is primarily metabolized via CYP2C19 and CYP3A4, and its AUC increases significantly with prolonged administration (by 159% at a 40 mg dose), suggesting that extended high-level exposure may elevate the risk of drug-related adverse events [18]. Further research is required to elucidate the clinical implications of this phenomenon.

Table 2 presents the top 20 PTs of adverse events associated with six PPIs, along with the corresponding number of reported cases (N). To assess the association between PPIs and these adverse events, multiple signal detection methods were employed.The Reporting ROR was calculated based on disproportionality analysis, where a positive signal is identified if the lower bound of the 95% confidence interval (ROR025) exceeds 1. The PRR assesses the signal strength, considering an event a potential positive signal if PRR ≥ 2, χ² ≥ 4, and case count ≥ 3. The Information Component (IC), estimated using a Bayesian statistical approach, measures the deviation of observed event occurrence from expected values, and a positive signal is confirmed if the lower bound of the 95% confidence interval (IC025) exceeds 0. The EBGM is used to evaluate signal intensity, with a positive signal determined when EB05 (the lower 5% confidence bound of EBGM) exceeds 1.If a PT meets all four positive signal criteria simultaneously, the adverse event is classified as a PPI-related adverse event and is marked as “Y” in the “positive signal” column for identification.

**Table 2:** The top 20 adverse reactions of six PPIs at the Preferred Term (PT) level.

	PT	N	ROR(95%CI)	PRR(χ2)	EBGM(95%CI)	IC025	positive signal
Esomeprazole							
1	Chronic kidney disease	16903	22.61(22.21-23.01)	21.23(250392.43)	16.48(16.20-16.78)	4.02	Y
2	Acute kidney injury	9559	5.52(5.41-5.64)	5.36(31683.98)	5.04(4.94-5.15)	2.30	Y
3	Renal failure	8896	5.45(5.33-5.57)	5.30(29051.34)	5.00(4.89-5.11)	2.29	Y
4	Gastrooesophageal reflux disease	7594	8.57(8.36-8.78)	8.35(44020.01)	7.56(7.38-7.74)	2.88	Y
5	Renal injury	5897	18.40(17.87-18.94)	18.01(75316.02)	14.50(14.09-14.93)	3.81	Y
6	End stage renal disease	5113	29.80(28.83-30.80)	29.25(98172.13)	20.86(20.17-21.56)	4.33	Y
7	Dyspepsia	4470	3.81(3.69-3.92)	3.76(8626.37)	3.62(3.51-3.73)	1.81	Y
8	Malaise	4145	0.72(0.70-0.74)	0.72(451.72)	0.72(0.70-0.75)	−0.51	
9	Pain	2867	0.35(0.34-0.36)	0.36(3414)	0.36(0.35-0.37)	−1.53	
10	Abdominal pain upper	2575	0.99(0.95-1.03)	0.99(0.17)	0.99(0.95-1.03)	−0.06	
11	Osteoporosis	2469	4.97(4.77-5.17)	4.93(7237.80)	4.67(4.48-4.86)	2.16	Y
12	Vomiting	2465	0.41(0.39-0.42)	0.41(2104.04)	0.41(0.40-0.43)	−1.33	
13	Nephrogenic anaemia	2285	72.24(68.13-76.60)	71.63(78256.90)	35.72(33.70-37.88)	5.06	Y
14	Nausea	2132	0.20(0.19-0.21)	0.21(6574.00)	0.21(0.20-0.22)	−2.23	
15	Abdominal discomfort	2091	1.00(0.96-1.04)	1.00(0.00)	1.00(0.96-1.04)	−0.06	
16	Tubulointerstitial nephritis	2052	9.79(9.34-10.25)	9.72(14086.89)	8.65(8.25-9.05)	3.04	Y
17	Diarrhoea	2014	0.24(0.23-0.25)	0.25(4815.59)	0.25(0.24-0.26)	−2.01	
18	Rebound acid hypersecretion	1918	122.44(113.67-131.90)	121.57(83266.29)	44.77(41.56-48.22)	5.37	Y
19	Dysphagia	1762	1.48(1.41-1.55)	1.47(266.00)	1.47(1.40-1.54)	0.48	
20	Death	1578	0.14(0.13-0.15)	0.14(8423.85)	0.14(0.14-0.15)	−2.86	
Lansoprazole							
1	Chronic kidney disease	12149	45.80(44.86-46.75)	40.01(385667.51)	33.43(32.75-34.13)	5.03	Y
2	Acute kidney injury	5829	9.56(9.31-9.83)	9.03(40106.82)	8.68(8.45-8.92)	3.08	Y
3	Renal failure	3649	6.13(5.92-6.33)	5.93(14609.58)	5.78(5.59-5.98)	2.48	Y
4	End stage renal disease	3528	52.76(50.81-54.78)	50.82(137141.37)	40.61(39.11-42.17)	5.27	Y
5	Renal injury	2954	23.45(22.56-24.37)	22.74(55147.15)	20.50(19.72-21.30)	4.30	Y
6	Tubulointerstitial nephritis	1392	18.22(17.24-19.25)	17.96(20456.25)	16.55(15.66-17.49)	3.95	Y
7	Renal impairment	1288	3.53(3.34-3.73)	3.50(2266.11)	3.45(3.27-3.65)	1.70	Y
8	Dialysis	1054	17.51(16.43-18.65)	17.32(14913.10)	16.00(15.02-17.05)	3.89	Y
9	Gastrooesophageal reflux disease	920	2.62(2.45-2.80)	2.60(901.50)	2.58(2.42-2.76)	1.27	Y
10	Death	869	0.22(0.20-0.23)	0.22(2428.82)	0.23(0.21-0.24)	−2.25	
11	Diarrhoea	857	0.29(0.27-0.31)	0.30(1474.91)	0.30(0.28-0.32)	−1.85	
12	Nephropathy	851	21.48(20.00-23.06)	21.30(14863.78)	19.32(17.99-20.74)	4.14	Y
13	Hyperchlorhydria	798	32.55(30.19-35.09)	32.28(20794.36)	27.88(25.86-30.06)	4.64	Y
14	Hyponatraemia	682	2.69(2.49-2.90)	2.68(708.58)	2.65(2.46-2.86)	1.29	Y
15	Proteinuria	619	7.77(7.17-8.42)	7.73(3492.59)	7.48(6.90-8.10)	2.77	Y
16	Hypertensive nephropathy	570	240.21(212.57-271.44)	238.76(61063.72)	108.57(96.08-122.69)	6.36	Y
17	Nephrogenic anaemia	556	28.01(25.62-30.61)	27.85(12614.39)	24.53(22.44-26.81)	4.43	Y
18	Diabetic nephropathy	538	69.40(62.90-76.58)	69.01(26718.57)	51.39(46.57-56.70)	5.41	Y
19	Nephrolithiasis	536	2.67(2.45-2.90)	2.66(548.09)	2.64(2.42-2.87)	1.27	Y
20	Rebound effect	524	14.12(12.92-15.43)	14.05(5930.89)	13.18(12.06-14.40)	3.56	Y
Dexlansoprazole							
1	Chronic kidney disease	2147	38.77(37.03-40.59)	33.96(66889.72)	32.98(31.50-34.52)	4.95	Y
2	Acute kidney injury	886	7.76(7.25-8.31)	7.41(4913.39)	7.36(6.88-7.88)	2.77	Y
3	Renal failure	748	6.91(6.42-7.44)	6.65(3594.18)	6.62(6.15-7.12)	2.61	Y
4	End stage renal disease	566	38.84(35.67-42.29)	37.57(19503.55)	36.37(33.40-39.60)	4.97	Y
5	Diarrhoea	364	0.70(0.63-0.77)	0.70(46.39)	0.70(0.63-0.78)	−0.66	
6	Renal injury	319	12.70(11.36-14.20)	12.48(3336.92)	12.35(11.05-13.81)	3.41	Y
7	Death	300	0.42(0.38-0.47)	0.43(231.38)	0.43(0.39-0.49)	−1.37	
8	Gastrooesophageal reflux disease	247	3.92(3.46-4.45)	3.88(528.77)	3.87(3.41-4.39)	1.75	Y
9	Nausea	208	0.32(0.28-0.36)	0.33(301.45)	0.33(0.28-0.37)	−1.81	
10	Tubulointerstitial nephritis	172	11.63(10.00-13.52)	11.52(1636.92)	11.41(9.81-13.27)	3.2	Y
11	Abdominal pain upper	164	0.99(0.85-1.16)	0.99(0.00)	0.99(0.85-1.16)	−0.23	
12	Dizziness	164	0.40(0.34-0.46)	0.40(148.02)	0.40(0.34-0.47)	−1.53	
13	Headache	145	0.28(0.24-0.33)	0.28(270.56)	0.28(0.24-0.33)	−2.05	
14	Dyspnoea	140	0.30(0.25-0.35)	0.30(229.10)	0.30(0.26-0.36)	−1.95	
15	Vomiting	138	0.36(0.30-0.42)	0.37(154.93)	0.37(0.31-0.43)	−1.69	
16	Rebound acid hypersecretion	129	49.87(41.77-59.52)	49.49(5867.53)	47.41(39.72-56.60)	4.87	Y
17	Abdominal pain	119	0.63(0.53-0.75)	0.63(25.67)	0.63(0.53-0.76)	−0.92	
18	Asthenia	107	0.34(0.28-0.42)	0.35(132.85)	0.35(0.29-0.42)	−1.79	
19	Pain	104	0.20(0.17-0.24)	0.21(329.46)	0.20(0.17-0.25)	−2.55	
20	Malaise	96	0.26(0.21-0.31)	0.26(201.37)	0.26(0.22-0.32)	−2.20	
Omeprazole							
1	Chronic kidney disease	4096	8.25(7.99-8.52)	8.04(23905.52)	7.64(7.40-7.89)	2.88	Y
2	Acute kidney injury	3204	3.34(3.23-3.46)	3.29(5018.39)	3.23(3.12-3.35)	1.64	Y
3	Renal failure	2149	2.35(2.25-2.46)	2.33(1616.98)	2.31(2.21-2.41)	1.14	Y
4	Diarrhoea	1972	0.45(0.43-0.47)	0.46(1270.41)	0.46(0.44-0.48)	−1.17	
5	Nausea	1782	0.33(0.31-0.34)	0.34(2402.14)	0.34(0.32-0.35)	−1.63	
6	Pain	1713	0.40(0.38-0.42)	0.41(1500.28)	0.41(0.39-0.43)	−1.35	
7	Hypomagnesaemia	1681	21.54(20.46-22.69)	21.30(28058.74)	18.50(17.57-19.49)	4.12	Y
8	Gastrooesophageal reflux disease	1359	2.63(2.50-2.78)	2.62(1338.46)	2.59(2.45-2.73)	1.29	Y
9	Vomiting	1321	0.42(0.40-0.44)	0.42(1054.47)	0.43(0.40-0.45)	−1.31	
10	Dizziness	1318	0.38(0.37-0.41)	0.39(1267.29)	0.39(0.37-0.42)	−1.42	
11	Dyspepsia	1290	2.02(1.91-2.14)	2.01(649.16)	2.00(1.89-2.11)	0.91	Y
12	Tubulointerstitial nephritis	1262	11.01(10.39-11.66)	10.92(10520.00)	10.17(9.60-10.77)	3.25	Y
13	Abdominal pain upper	1238	0.91(0.86-0.96)	0.91(10.71)	0.91(0.86-0.96)	−0.22	
14	Headache	1219	0.28(0.27-0.30)	0.29(2205.77)	0.29(0.27-0.31)	−1.87	
15	Dyspnoea	1208	0.31(0.29-0.33)	0.32(1820.42)	0.32(0.30-0.34)	−1.73	
16	Hyperchlorhydria	1104	32.47(30.40-34.68)	32.21(26906.33)	26.14(24.48-27.93)	4.58	Y
17	End stage renal disease	1100	9.16(8.26-9.74)	9.10(7431.57)	8.58(8.07-9.13)	3.00	Y
18	Rebound effect	1076	21.24(19.91-22.66)	21.09(17784.93)	18.34(17.20-19.57)	4.08	Y
19	Renal injury	1055	5.14(4.84-5.47)	5.11(3367.23)	4.96(4.66-5.28)	2.21	Y
20	Hypocalcaemia	1029	8.73(8.20-9.30)	8.67(6562.89)	8.20(7.70-8.74)	2.93	Y
Pantoprazole							
1	Chronic kidney disease	7739	22.39(21.85-22.94)	20.84(130990.03)	18.71(18.26-19.17)	4.19	Y
2	Acute kidney injury	4354	6.06(5.88-6.25)	5.86(17081.60)	5.70(5.52-5.88)	2.46	Y
3	Renal failure	2348	3.37(3.23-3.51)	3.31(3745.90)	3.27(3.14-3.41)	1.65	Y
4	End stage renal disease	2311	27.41(26.22-28.65)	26.84(49825.88)	23.37(22.36-24.43)	4.47	Y
5	Renal injury	1226	7.84(7.40-8.30)	7.76(6925.20)	7.47(7.06-7.92)	2.81	Y
6	Nausea	1186	0.28(0.27-0.30)	0.29(2119.93)	0.29(0.28-0.31)	−1.86	
7	Gastrooesophageal reflux disease	1144	2.87(2.71-3.05)	2.85(1359.93)	2.82(2.66-2.99)	1.41	Y
8	Diarrhoea	1127	0.34(0.32-0.36)	0.34(1466.32)	0.34(0.32-0.36)	−1.63	
9	Tubulointerstitial nephritis	1113	12.50(11.76-13.29)	12.38(10879.69)	11.62(10.94-12.36)	3.44	Y
10	Hypomagnesaemia	917	14.17(13.25-15.17)	14.06(10299.38)	13.08(12.23-14.00)	3.59	Y
11	Dyspnoea	916	0.31(0.29-0.33)	0.31(1424.50)	0.31(0.29-0.33)	−1.77	
12	Death	910	0.20(0.19-0.21)	0.21(2896.65)	0.21(0.19-0.22)	−2.37	
13	Vomiting	873	0.36(0.34-0.38)	0.36(993.90)	0.36(0.34-0.39)	−1.55	
14	Fatigue	786	0.19(0.18-0.20)	0.20(2679.15)	0.20(0.18-0.21)	−2.44	
15	Dizziness	784	0.30(0.28-0.32)	0.30(1288.36)	0.30(0.28-0.33)	−1.82	
16	Headache	714	0.21(0.20-0.23)	0.22(2048.75)	0.22(0.20-0.24)	−2.29	
17	Malaise	691	0.29(0.27-0.32)	0.30(1165.67)	0.30(0.28-0.32)	−1.85	
18	Hypocalcaemia	679	7.31(6.77-7.89)	7.27(3526.21)	7.01(6.50-7.58)	2.68	Y
19	Pruritus	667	0.36(0.33-0.39)	0.36(757.23)	0.36(0.34-0.39)	−1.57	
20	Abdominal pain	664	0.55(0.51-0.59)	0.55(239.78)	0.55(0.51-0.60)	−0.96	
Rabeprazole							
1	Chronic kidney disease	487	12.54(11.44-13.74)	12.00(4899.08)	11.93(10.89-13.07)	3.41	Y
2	Renal failure	475	6.99(6.38-7.67)	6.72(2321.68)	6.70(6.11-7.35)	2.59	Y
3	Acute kidney injury	211	2.84(2.48-3.25)	2.80(245.66)	2.80(2.44-3.21)	1.27	Y
4	Pruritus	138	0.76(0.64-0.90)	0.76(10.27)	0.76(0.65-0.90)	−0.63	
5	Diarrhoea	129	0.39(0.33-0.47)	0.40(120.86)	0.40(0.34-0.47)	−1.58	
6	Rash	116	0.53(0.45-0.64)	0.34(46.51)	0.54(0.45-0.65)	−1.15	
7	Dizziness	109	0.42(0.35-0.51)	0.43(85.35)	0.43(0.35-0.52)	−1.49	
8	Nausea	107	0.26(0.21-0.31)	0.27(223.14)	0.27(0.22-0.32)	−2.17	
9	Headache	102	0.31(0.26-0.38)	0.32(153.56)	0.32(0.26-0.39)	−1.93	
10	Hypomagnesaemia	91	13.35(10.86-16.43)	13.2(1023.45)	13.16(10.70-16.18)	3.24	Y
11	Hyponatraemia	90	3.14(2.55-3.86)	3.12(129.93)	3.12(2.53-3.84)	1.30	Y
12	Tubulointerstitial nephritis	88	9.43(7.64-11.64)	9.36(654.00)	9.31(7.55-11.49)	2.78	Y
13	Gastrooesophageal reflux disease	86	2.16(1.75-2.67)	2.15(53.18)	2.15(1.74-2.66)	0.77	
14	Dyspnoea	84	0.29(0.23-0.35)	0.29(149.02)	0.29(0.23-0.36)	−2.08	
15	Abdominal pain	80	0.68(0.54-0.84)	0.68(12.33)	0.68(0.54-0.85)	−0.88	
16	Urticaria	79	0.96(0.77-1.20)	0.96(0.14)	0.96(0.77-1.20)	−0.38	
17	Vomiting	79	0.33(0.26-0.41)	0.33(107.09)	0.33(0.27-0.42)	−1.89	
18	Dyspepsia	76	1.55(1.24-1.94)	1.55(14.68)	1.54(1.23-1.94)	0.29	
19	Malaise	73	0.32(0.25-0.40)	0.32(107.64)	0.32(0.25-0.40)	−1.96	
20	Arthralgia	70	0.33(0.26-0.42)	0.34(93.57)	0.34(0.27-0.43)	−1.90	

According to Table 2, PPIs exhibit positive signals for multiple adverse events (AEs) in both the renal system and gastrointestinal system, with renal-related adverse events being particularly prominent. All six PPIs demonstrate statistically significant positive signals for CKD, AKI, end-stage renal disease (ESRD), and tubulointerstitial nephritis (TIN). In addition to reaffirming well-established adverse reactions such as acute interstitial nephritis, acute kidney injury, chronic kidney disease, and hypomagnesaemia, this study identified several emerging safety signals. Notably, significant signals were observed for hypertensive nephropathy and diabetic nephropathy, suggesting that PPI use may accelerate renal deterioration among patients with underlying cardiometabolic comorbidities. We also detected a clear signal for nephrogenic anaemia, an adverse reaction rarely reported in previous pharmacovigilance studies, warranting confirmation in prospective cohorts.

Furthermore, prominent signals were identified for electrolyte disturbances, including hyponatraemia and hypocalcaemia, which, despite being supported mainly by isolated case reports, may carry important clinical implications. Importantly, our analysis quantified pharmacovigilance-level signals for rebound acid hypersecretion, rebound effect, and hyperchlorhydria. Although these phenomena have been clinically described, large-scale real-world evidence has been limited. Our findings suggest that acid overproduction following PPI discontinuation may be more common than previously recognized.

Among these, Lansoprazole exhibits the most pronounced positive signals for CKD (ROR = 45.80, IC025 = 5.03) and ESRD (ROR = 52.76, IC025 = 5.27), followed by Dexlansoprazole, which shows significant signals for CKD (ROR = 38.77, IC025 = 4.95) and ESRD (ROR = 38.84, IC025 = 4.97), indicating a potential risk of renal failure with long-term use. In addition, Lansoprazole also presents a notable positive signal for nephrogenic anaemia (ROR = 28.01, IC025 = 4.43). Notably, Lansoprazole demonstrates an exceptionally strong signal for hypertensive nephropathy (ROR = 240.21, IC025 = 6.36), which requires further clinical attention.

Meanwhile, Esomeprazole also exhibits an extremely strong positive signal for nephrogenic anaemia (ROR = 72.24, IC025 = 5.06), which is consistent with Wang’s study results [19]. Furthermore, this drug shows the highest signal strength for rebound acid hypersecretion (ROR = 122.44, IC025 = 5.37), suggesting a significant risk of acid rebound upon discontinuation. However, this phenomenon has not been observed with Dexlansoprazole, Pantoprazole, or Rabeprazole.

In terms of electrolyte metabolism disorders, hypomagnesemia, hyponatremia, and hypocalcemia exhibit positive signals across different PPIs, indicating that long-term use may lead to electrolyte imbalances. Omeprazole (ROR = 21.54, IC025 = 4.12), Pantoprazole (ROR = 14.17, IC025 = 3.59), and Rabeprazole (ROR = 13.35, IC025 = 3.24) show strong positive signals for hypomagnesemia, which may be related to PPIs interfering with magnesium absorption and reabsorption in the small intestine and renal tubules. Hyponatremia is mainly observed with Lansoprazole (ROR = 2.69, IC025 = 1.29) and Rabeprazole (ROR = 3.14, IC025 = 1.30), suggesting that these drugs may affect renal water-electrolyte regulation, leading to decreased serum sodium levels. Hypocalcemia is primarily associated with Omeprazole (ROR = 8.73, IC025 = 2.93) and Pantoprazole (ROR = 7.31, IC025 = 2.68), indicating a potential reduction in calcium absorption due to decreased gastric acid secretion, which may contribute to bone metabolism disorders, osteoporosis, and an increased risk of fractures. Therefore, for patients on long-term PPI therapy, particularly elderly individuals with impaired renal function, Regular monitoring of serum biochemical parameters and follow-up are recommended to minimize the potential risk of electrolyte disturbances.

**Fig 3:**
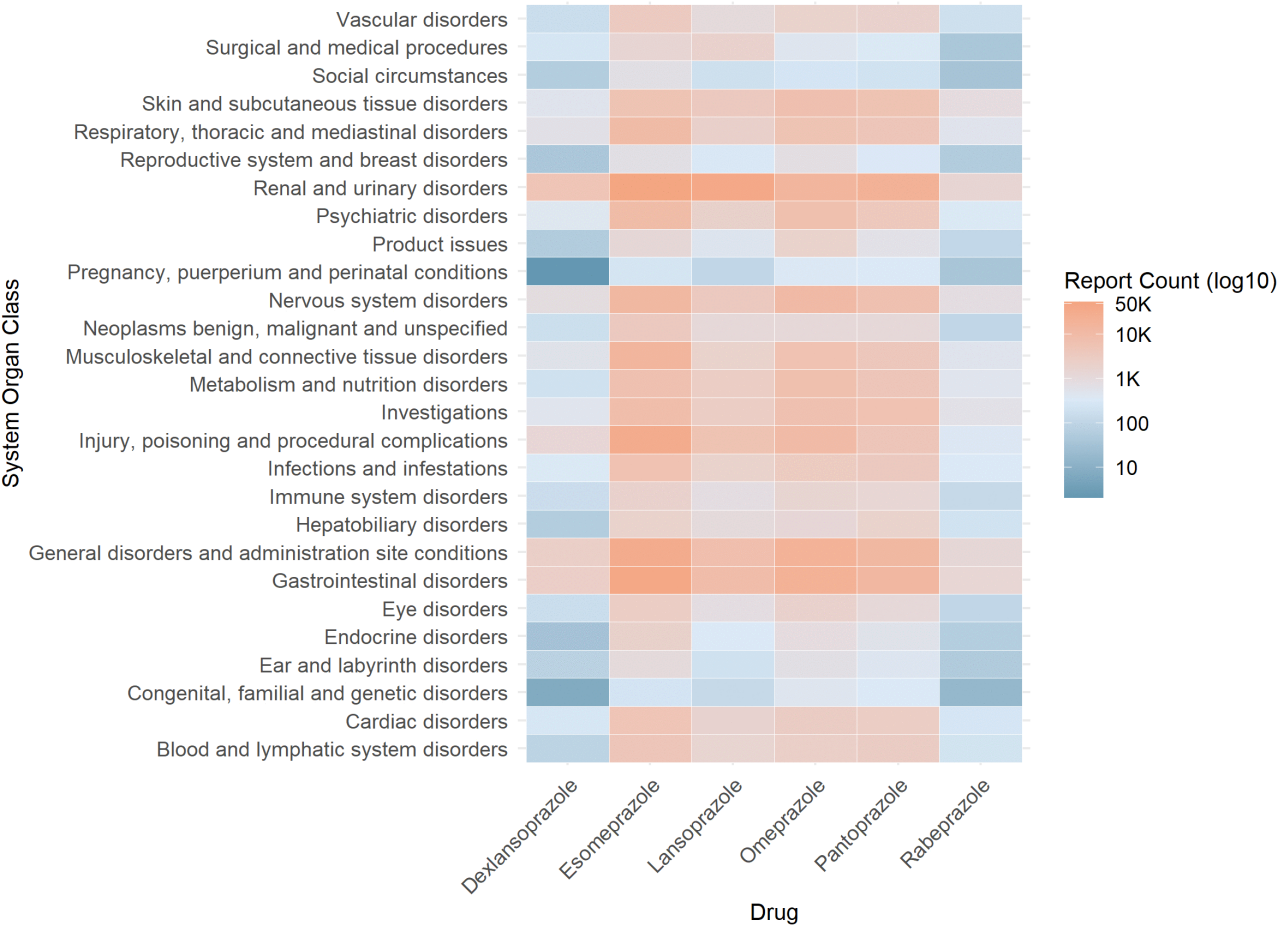
Heatmap of the adverse reactions of six types of PPIs classified according to the System Organ Class (SOC). The y-axis lists SOC categories, and the x-axis lists PPIs. Color intensity corresponds to the log10-transformed number of reports (per the legend scale), providing an overview of reporting distribution across organ systems.

All adverse events associated with PTs were categorized based on the SOC classification. After excluding the categories of Social Circumstances and Product Issues categories, the results were visualized using a heatmap. As illustrated in Figure (Fig 3), the distribution of PPI-related adverse events at the SOC level aligns with the findings at the PT level, predominantly affecting the Renal and Urinary Disorders and Gastrointestinal Disorders, followed by General Disorders and Administration Site Conditions and systemic metabolic abnormalities such as electrolyte Imbalance and systemic metabolic disorders. The use of PPIs in pregnant women appears relatively safe, aligning with the FDA labeling risk assessment and classification standards.

This study leveraged large-scale spontaneous reporting data from multiple countries and regions, providing strong external validity to the general population. The major safety signals detected—such as renal adverse events, electrolyte disturbances, and rebound acid hypersecretion—are supported by previous studies and well-established pharmacological mechanisms, indicating broad applicability. However, variations in prescribing preferences and drug-use patterns across regions limit the feasibility of detailed stratified analyses. Therefore, validation using domestic real-world databases, health insurance data, and prospective cohort studies remains essential to ensure the applicability of these findings to local clinical practice. Future high-quality studies are warranted to further strengthen the evidence base for these safety signals.

## 4. Discussion

PPIs have been associated with long-term off-label use, overuse, and prolonged administration in clinical practice [20], with their potential adverse effects drawing significant attention [9], particularly those affecting the renal/urinary and gastrointestinal systems. A prescription analysis study conducted across 45 hospitals in four Chinese cities revealed a substantial prevalence of inappropriate PPI prescriptions, especially among patients without clear indications for long-term excessive use [21]. An Italian study involving over 2000 patients from 27 nursing homes demonstrated that while 45.6% of patients were prescribed PPIs, only 50.7% of these prescriptions were based on evidence-based indications (such as peptic ulcers and Helicobacter pylori infection) [20]. Notably, the misuse of PPIs is particularly pronounced in the context of polypharmacy. These irrational prescribing patterns further exacerbate the risk of drug-related adverse events.

The findings of this study, in conjunction with existing evidence, necessitate heightened vigilance regarding the clinical use of PPIs, as long-term administration may significantly elevate the risk of adverse effects [22]. This concern is particularly evident in the renal and urinary systems, where significant associations between AKI or CKD events and PPIs have been reported since the early use of omeprazole, dating back to the 1990s [23,24]. Previous pharmacovigilance studies based on the FAERS database have shown significant signal associations between PPIs and various renal adverse outcomes. Jain et al. applied data-mining algorithms such as PRR and ROR, and identified marked disproportional reporting of acute kidney injury, chronic kidney disease, renal failure, and end-stage renal disease among PPI users, suggesting that the potential nephrotoxicity associated with PPI exposure warrants careful attention.An observational study shows a direct association between PPIs and CKD [25], which remains consistent even when baseline PPI users are compared directly with H2 receptor antagonist users [26]. Another study based on the FAERS database indicates that dexlansoprazole has the strongest association with both CKD and AKI among the six PPIs analyzed [27], and AKI results in more deaths, life-threatening events, hospitalizations, and disabilities compared to CKD. Our statistical analysis reveals strong associations for both lansoprazole and dexlansoprazole. Differences in data cleaning and filtering methods may account for variations, as some studies exclude non-professional reports but reach similar conclusions. A longitudinal follow-up study confirms a 50% increased risk of CKD development among PPI users versus non-users (HR = 1.50, 95% CI:1.14–1.96), with risk increasing proportionally to treatment duration [25]. Further research demonstrates that PPI users remain at elevated risk for CKD and end-stage renal disease even without experiencing AKI, suggesting that PPIs might exacerbate renal pathology through mechanisms independent of acute injury [22]. These findings argue against simplistic causal attributions and underscore the need for careful risk-benefit assessment in PPI therapy.

PPIs significantly interfere with the metabolism and absorption of ions such as calcium, magnesium, and iron by inhibiting gastric acid secretion and affecting renal excretion, thereby reducing their bioavailability. PPIs inhibit the H ⁺ /K ⁺ -ATPase in gastric parietal cells, markedly reducing gastric acid secretion and leading to decreased gastric acidity [28]. This low-acid environment diminishes the absorption of calcium, magnesium, and iron ions in the intestine. Additionally, PPIs can indirectly affect renal function by inhibiting gastrin secretion, which subsequently reduces the expression of parathyroid hormone-like hormone (PTHLH), further disrupting the metabolism of calcium, magnesium, and iron.

Gastric acid secretion facilitates calcium absorption. Prolonged use of PPIs can reduce the absorption of calcium ions from food, thereby decreasing bone mineral density and increasing the risk of osteoporosis and secondary fractures [28]. A large-scale study demonstrated that long-term PPI use [29], particularly with escalating doses or extended duration, is positively correlated with an increased risk of hip fractures, especially in men. Another study corroborated these concerns, prompting the FDA to include this warning in drug labels in 2010 [30].

Long-term PPI use has been causally linked to hypomagnesemia [31,32], with case reports and studies indicating that the specific mechanisms remain unclear. These mechanisms may involve increased cellular uptake of magnesium and reduced gastrointestinal absorption of magnesium, potentially leading to severe symptoms such as fatigue, muscle weakness, and arrhythmias [33]. Notably, the article further suggests that dietary interventions targeting the gut microbiota could serve as an effective treatment strategy [34]. Danziger et al [35]. demonstrated that the combined use of PPIs and diuretics increases the risk of hypomagnesemia in patients, necessitating long-term monitoring of serum magnesium levels in high-risk individuals and minimizing unnecessary polypharmacy.

Additionally, the lack of gastric acid can impair the dissolution and absorption of non-heme iron, and iron deficiency may further increase the risk of renal anemia [36]. Another study indicates that long-term PPI use, which suppresses gastric acid, is positively correlated with low vitamin B12 levels [37], thereby elevating the risk of related diseases. This study detected positive signals for renal anemia in both esomeprazole and lansoprazole, further emphasizing the importance of regular monitoring of relevant indicators during long-term PPI use to prevent associated complications.

During the treatment of gastric acid suppression with PPIs, instances of acid rebound and unresolved symptoms of GERD may occur. Although the use of PPIs in GERD treatment is widely recognized, their efficacy in refractory GERD is limited. Studies suggest that this refractoriness may be attributed to multiple factors [38], including rapid PPI metabolism, poor medication adherence, nocturnal acid breakthrough, non-acidic reflux, or functional heartburn. It is essential to first evaluate the appropriateness of PPI use, further clarify the underlying etiology, and adjust the treatment plan accordingly. Additionally, gradual dose reduction during discontinuation is crucial to avoid acid rebound caused by feedback stimulation of gastric parietal cells, which may lead to symptoms such as heartburn, acid regurgitation, and dyspepsia [39].

In summary, PPIs should be prescribed or sold only for clear indications, at low doses, and for short durations. PPI treatment should be discontinued in users without indications or with unclear indications, and even those with indications should avoid long-term use unless necessary. Patients with primary kidney disease should exercise particular caution when using PPIs, and long-term users should undergo regular renal function monitoring [40]. Long-term PPI use without indications should be avoided, and dose reduction should be considered when appropriate to mitigate potential mortality risks [41].

In our database research, rabeprazole exhibited the lowest number of adverse reaction reports but the highest mortality rate, whereas esomeprazole had the highest number of cases but a lower mortality rate. This phenomenon suggests the potential existence of drug selection bias: high-risk patients may be more inclined to use specific PPIs, such as rabeprazole for end-stage patients. A study indicated that rabeprazole does not require dose adjustment for patients with stable, end-stage renal failure; however, due to the small sample size, further clinical trials are still needed [42].

Bias exists in the information collection of databases, where reports on specific drugs or biological products cannot confirm whether adverse reactions are caused by the drug in question; they merely represent the reporter’s PS [43]. For any given adverse drug event (ADE) report, the ADE signal calculated based on disproportionality analysis indicates a statistical correlation between the target drug and the target ADE, but not a biological one. This does not prove a definitive causal relationship, necessitating further in-depth investigation through clinical trials and prospective real-world studies. Additionally, the regional distribution of FAERS is predominantly centered in the United States, with limited data from Asia and Africa, resulting in insufficient representativeness. Given the variation in prescribing habits and drug-use preferences across different countries and regions, fine-grained stratified analyses based solely on spontaneous reports are difficult to achieve. The reporting of case data is primarily consumer-driven, with voluntary and self-reported submissions lacking professionalism and review, inevitably leading to issues such as underreporting, overreporting, inconsistent or missing information [44]. The high proportion of consumer-submitted reports may further introduce bias, as consumers tend to report more subjective or symptom-driven reactions, while clinically silent or laboratory-based events are less likely to be captured. In addition, consumers may be more influenced by media attention or personal perception of risk, which can selectively amplify certain adverse events. The limitations of database analysis underscore the need for more clinical research to validate the robustness and clinical significance of the observed signals.

## 5. Conclusions

This study systematically analyzed PPI-associated adverse events using data from the FAERS database, aiming to assess potential risks and associations, thereby providing scientific evidence and theoretical support for clinical practice.

## Supporting information

S1 Table SOC-level adverse reactions of six PPIs. (XLSX)

S2 Table PPI adverse reaction reports(2004–2024). (XLSX)

S3 Table Adverse reaction dataset stratified by time. (XLSX)

## References

[1] Baik SH, Fung K-W, McDonald CJ. The mortality risk of proton pump inhibitors in 1.9 million us seniors: an extended cox survival analysis. Clin Gastroenterol Hepatol. 2022;20(4):e671–81. doi: 10.1016/j.cgh.2021.01.014 33453399 PMC12381940

[2] Yu L-Y, Sun L-N, Zhang X-H, Li Y-Q, Yu L, Yuan Z-Q-Y, et al. A review of the novel application and potential adverse effects of proton pump inhibitors. Adv Ther. 2017;34(5):1070–86. doi: 10.1007/s12325-017-0532-9 28429247 PMC5427147

[3] Bhatt DL, Scheiman J, Abraham NS, Antman EM, Chan FK, Furberg CD, et al. ACCF/ACG/AHA 2008 expert consensus document on reducing the gastrointestinal risks of antiplatelet therapy and NSAID use: a report of the American college of cardiology foundation task force on clinical expert consensus documents. Circulation. 2008;118(18):1894–909. 18836135 10.1161/CIRCULATIONAHA.108.191087

[4] Wu Y, Hu Y, You P, Chi Y-J, Zhou J-H, Zhang Y-Y, et al. Study of clinical and genetic risk factors for aspirin-induced gastric mucosal injury. Chin Med J (Engl). 2016;129(2):174–80. doi: 10.4103/0366-6999.173480 26830988 PMC4799544

[5] Moayyedi P, Eikelboom JW, Bosch J, Connolly SJ, Dyal L, Shestakovska O, et al. Safety of proton pump inhibitors based on a large, multi-year, randomized trial of patients receiving rivaroxaban or aspirin. Gastroenterology. 2019;157(3):682-691.e2. doi: 10.1053/j.gastro.2019.05.056 31152740

[6] Vaduganathan M, Cannon CP, Cryer BL, Liu Y, Hsieh WH, Doros G, et al. Efficacy and safety of proton-pump inhibitors in high-risk cardiovascular subsets of the COGENT trial. Am J Med. 2016;129(9):1002–5. 27143321 10.1016/j.amjmed.2016.03.042

[7] Alshamsi F, Belley-Cote E, Cook D, Almenawer SA, Alqahtani Z, Perri D, et al. Efficacy and safety of proton pump inhibitors for stress ulcer prophylaxis in critically ill patients: a systematic review and meta-analysis of randomized trials. Crit Care. 2016;20(1):120. 27142116 10.1186/s13054-016-1305-6 PMC4855320

[8] Shin JM, Cho YM, Sachs G. Chemistry of covalent inhibition of the gastric (H , K )-ATPase by proton pump inhibitors. J Am Chem Soc. 2004;126(25):7800–11. 15212527 10.1021/ja049607w

[9] Haastrup PF, Thompson W, Søndergaard J, Jarbøl DE. Side effects of long-term proton pump inhibitor use: a review. Basic Clin Pharmacol Toxicol. 2018;123(2):114–21. doi: 10.1111/bcpt.13023 29658189

[10] Aseeri M, Schroeder T, Kramer J, Zackula R. Gastric acid suppression by proton pump inhibitors as a risk factor for clostridium difficile-associated diarrhea in hospitalized patients. Am J Gastroenterol. 2008;103(9):2308–13. doi: 10.1111/j.1572-0241.2008.01975.x 18702653

[11] Yang Y-X, Metz DC. Safety of proton pump inhibitor exposure. Gastroenterology. 2010;139(4):1115–27. doi: 10.1053/j.gastro.2010.08.023 20727892

[12] Chen G, Ning L-J, Qin Y, Zhao B, Mei D, Li X-M. Acute kidney injury following the use of different proton pump inhibitor regimens: A real-world analysis of post-marketing surveillance data. J Gastroenterol Hepatol. 2021;36(1):156–62. doi: 10.1111/jgh.15151 32542684

[13] Zhai Y, Ye X, Hu F, Xu J, Guo X, Lin Z, et al. Updated Insights on Cardiac and Vascular Risks of Proton Pump Inhibitors: A Real-World Pharmacovigilance Study. Front Cardiovasc Med. 2022;9:767987. doi: 10.3389/fcvm.2022.767987 35282344 PMC8913586

[14] Sun Y, Zhang A, Zuo M, Chen J, Zhu L. A pharmacovigilance study of association between proton-pump inhibitors and rhabdomyolysis event based on FAERS database. J Gastroenterol Hepatol. 2024;39(2):289–96. doi: 10.1111/jgh.16411 37961012

[15] Zhang Y-J, Duan D-D, Tian Q-Y, Wang C-E, Wei S-X. A pharmacovigilance study of the association between proton pump inhibitors and tumor adverse events based on the FDA adverse event reporting system database. Front Pharmacol. 2024;15:1524903. doi: 10.3389/fphar.2024.1524903 39749203 PMC11694325

[16] Dong Y, Wang Y, Lan X, Zeng H. A study on the pharmacovigilance of various SGLT-2 inhibitors. Front Med (Lausanne). 2025;11:1515847. 39882521 10.3389/fmed.2024.1515847 PMC11774954

[17] Pan H, Shi X, Jiang Y, Wu J, Shen L. Analyzing the adverse events of NK-1 receptor antagonists: a pharmacovigilance study from the FAERS database. Sci Rep. 2024;14(1):31201. 39732926 10.1038/s41598-024-82575-5 PMC11682360

[18] Scott LJ, Dunn CJ, Mallarkey G, Sharpe M. Esomeprazole: a review of its use in the management of acid-related disorders. Drugs. 2000. 10.2165/00003495-200262100-00006 12093317

[19] Wang B, Huang S, Li S, Deng Y, Li Z, Wang Y, et al. Safety assessment of esomeprazole: real-world adverse event signal mining and analysis based on FAERS database. Expert Opin Drug Saf. 2025;:1–9. doi: 10.1080/14740338.2025.2473636 40019211

[20] Pasina L, Novella A, Elli C, Nobili A, Ianes A. Overuse of proton pump inhibitors in nursing homes: An Italian multicenter observational study. Pharmacoepidemiol Drug Saf. 2020;29(4):461–6. doi: 10.1002/pds.4963 31990131

[21] Ying J, Li L-C, Wu C-Y, Yu Z-W, Kan L-D. The status of proton pump inhibitor use: a prescription survey of 45 hospitals in China. Rev Esp Enferm Dig. 2019;111(10):738–43. doi: 10.17235/reed.2019.6155/2019 31373505

[22] Xie Y, Bowe B, Li T, Xian H, Yan Y, Al-Aly Z. Long-term kidney outcomes among users of proton pump inhibitors without intervening acute kidney injury. Kidney Int. 2017;91(6):1482–94. doi: 10.1016/j.kint.2016.12.021 28237709

[23] Ruffenach SJ, Siskind MS, Lien YH. Acute interstitial nephritis due to omeprazole. Am J Med. 1992;93(4):472–3. doi: 10.1016/0002-9343(92)90181-a 1341422

[24] Christensen PB, Albertsen KE, Jensen P. Renal failure after omeprazole. Lancet. 1993;341(8836):55. doi: 10.1016/0140-6736(93)92531-w 8093302

[25] Lazarus B, Chen Y, Wilson FP, Sang Y, Chang AR, Coresh J, et al. Proton pump inhibitor use and the risk of chronic kidney disease. JAMA Intern Med. 2016;176(2):238–46. doi: 10.1001/jamainternmed.2015.7193 26752337 PMC4772730

[26] Jain D, Sharma G, Kumar A. Adverse effects of proton pump inhibitors (PPIs) on the renal system using data mining algorithms (DMAs). Expert Opin Drug Saf. 2023;22(8):741–52. doi: 10.1080/14740338.2023.2189698 36888736

[27] Wu B, Li D, Xu T, Luo M, He Z, Li Y. Proton pump inhibitors associated acute kidney injury and chronic kidney disease: data mining of US FDA adverse event reporting system. Sci Rep. 2021;11(1):3690. doi: 10.1038/s41598-021-83099-y 33574396 PMC7878877

[28] Ito T, Jensen RT. Association of long-term proton pump inhibitor therapy with bone fractures and effects on absorption of calcium, vitamin B12, iron, and magnesium. Curr Gastroenterol Rep. 2010;12(6):448–57. doi: 10.1007/s11894-010-0141-0 20882439 PMC2974811

[29] Yang Y-X, Lewis JD, Epstein S, Metz DC. Long-term proton pump inhibitor therapy and risk of hip fracture. JAMA. 2006;296(24):2947–53. doi: 10.1001/jama.296.24.2947 17190895

[30] Vestergaard P, Rejnmark L, Mosekilde L. Proton pump inhibitors, histamine H2 receptor antagonists, and other antacid medications and the risk of fracture. Calcif Tissue Int. 2006;79(2):76–83. doi: 10.1007/s00223-006-0021-7 16927047

[31] Mackay JD, Bladon PT. Hypomagnesaemia due to proton-pump inhibitor therapy: a clinical case series. QJM. 2010;103(6):387–95. doi: 10.1093/qjmed/hcq021 20378675

[32] Cundy T, Mackay J. Proton pump inhibitors and severe hypomagnesaemia. Curr Opin Gastroenterol. 2011;27(2):180–5. doi: 10.1097/MOG.0b013e32833ff5d6 20856115

[33] Liao S, Gan L, Mei Z. Does the use of proton pump inhibitors increase the risk of hypomagnesemia: An updated systematic review and meta-analysis. Medicine (Baltimore). 2019;98(13):e15011. doi: 10.1097/MD.0000000000015011 30921222 PMC6456119

[34] Gommers LMM, Hoenderop JGJ, de Baaij JHF. Mechanisms of proton pump inhibitor-induced hypomagnesemia. Acta Physiol (Oxf). 2022;235(4):e13846. doi: 10.1111/apha.13846 35652564 PMC9539870

[35] Danziger J, William JH, Scott DJ, Lee J, Lehman L, Mark RG, et al. Proton-pump inhibitor use is associated with low serum magnesium concentrations. Kidney Int. 2013;83(4):692–9. doi: 10.1038/ki.2012.452 23325090 PMC5682024

[36] Shikata T, Sasaki N, Ueda M, Kimura T, Itohara K, Sugahara M, et al. Use of proton pump inhibitors is associated with anemia in cardiovascular outpatients. Circ J. 2015;79(1):193–200. doi: 10.1253/circj.CJ-14-0582 25392070

[37] Dharmarajan TS, Kanagala MR, Murakonda P, Lebelt AS, Norkus EP. Do acid-lowering agents affect vitamin B12 status in older adults?. J Am Med Dir Assoc. 2008;9(3):162–7. doi: 10.1016/j.jamda.2007.10.004 18294598

[38] Yadlapati R, DeLay K. Proton pump inhibitor-refractory gastroesophageal reflux disease. Med Clin North Am. 2019;103(1):15–27. 30466671 10.1016/j.mcna.2018.08.002 PMC6260943

[39] Namikawa K, Björnsson ES. Rebound acid hypersecretion after withdrawal of long-term proton pump inhibitor (PPI) treatment-are PPIs addictive?. Int J Mol Sci. 2024;25(10):5459. 38791497 10.3390/ijms25105459 PMC11122117

[40] Al-Aly Z, Maddukuri G, Xie Y. Proton pump inhibitors and the kidney: implications of current evidence for clinical practice and when and how to deprescribe. Am J Kidney Dis. 2020;75:497–500. 31606235 10.1053/j.ajkd.2019.07.012

[41] Xie Y, Bowe B, Li T, Xian H, Yan Y, Al-Aly Z. Risk of death among users of Proton Pump Inhibitors: a longitudinal observational cohort study of United States veterans. BMJ Open. 2017;7(6):e015735. doi: 10.1136/bmjopen-2016-015735 28676480 PMC5642790

[42] Keane WF, Swan SK, Grimes I, Humphries TJ. Rabeprazole: pharmacokinetics and tolerability in patients with stable, end-stage renal failure. J Clin Pharmacol. 1999;39(9):927–33. doi: 10.1177/00912709922008542 10471983

[43] Weiss-Smith S, Deshpande G, Chung S, Gogolak V. The FDA drug safety surveillance program: adverse event reporting trends. Arch Intern Med. 2011;171(6):591–3. 21444854 10.1001/archinternmed.2011.89

[44] Palleria C, Leporini C, Chimirri S, Marrazzo G, Sacchetta S, Bruno L, et al. Limitations and obstacles of the spontaneous adverse drugs reactions reporting: two “challenging” case reports. J Pharmacol Pharmacother. 2013;4(Suppl 1):S66-72. 10.4103/0976-500X.120955 PMC3853673 24347986

